# Longitudinal psychosocial burden, quality of life, and digital supportive care preferences before and after CAR T-cell therapy: a prospective single-center observational study

**DOI:** 10.1186/s12885-026-17245-y

**Published:** 2026-10-27

**Authors:** Anna Fleischer, Sabine-Anna Bergert, Patrick-Pascal Strunz, Barbara Huber, Nadine Schassberger, Miriam Benz, Elisabeth Jentschke, Florian Eisele, Yoko Tamamushi, Hermann Einsele, Leo Rasche, Michael Hudecek, Johannes Duell, Max Topp, Imad Maatouk

**Affiliations:** 1 Department of Internal Medicine II, Psychosomatic Medicine, University Hospital Würzburg, Würzburg, Germany; 2 Department of Internal Medicine II, Rheumatology, University Hospital Würzburg, Würzburg, Germany; 3 Department of Internal Medicine II, Hematology and Oncology, University Hospital Würzburg, Würzburg, Germany

**Keywords:** CAR T-cell therapy, Patient-reported outcomes, Quality of life, Psycho-oncology, Digital health, Supportive care

## Abstract

**Background:**

Prospective studies of CAR T-cell therapy have described changes in patient-reported outcomes, but these are rarely examined alongside supportive care preferences and patients’ own accounts. We examined these components together across the treatment course.

**Methods:**

This prospective single-center study included adults scheduled for CAR T-cell therapy at a German tertiary hospital. Of 52 patients in the screening/recruitment log, 32 who completed at least three of four assessments formed the longitudinal cohort. Assessments occurred before treatment (T0), during post-infusion hospitalization (T1), at or around discharge (T2), and seven to eight weeks after discharge (T3). Standardized measures assessed quality of life, anxiety/depression, loneliness, fear of progression, somatic symptoms, and social support. Unadjusted generalized estimating equations examined longitudinal change; supportive care items were summarized descriptively and open-ended responses analyzed thematically.

**Results:**

The cohort included 17 male and 15 female participants; median age was 61.0 years (IQR 53.0-65.5), and 23/32 (71.9%) had multiple myeloma. Global health status/quality of life showed an overall time effect (p = 0.001), with the lowest mean at T2 and recovery by T3. Physical functioning was nominally lower at T2 (β = -14.450, p = 0.046); fatigue was nominally higher at T1 and T2 (β = 11.990, p = 0.015; β = 15.574, p = 0.008), and the QLQ-C30 summary score lower at T1 and T2 (β = -7.772, p = 0.006; β = -6.954, p = 0.035). Loneliness was nominally higher at T1 (β = 0.795, p = 0.009), whereas PHQ-4 scores changed little. Across timepoints, 60.0% to 70.0% considered post-discharge psycho-oncological support useful and 66.7% to 79.3% considered peer contact useful. Five themes concerned treatment uncertainty, physical decline, multidimensional support needs, accessible individualized digital support, and incomplete treatment understanding.

**Conclusions:**

Patient-reported physical and functional burden was greatest during hospitalization and around discharge and improved by seven to eight weeks after discharge. Brief anxiety/depression scores captured only part of participants’ support needs. The findings can inform co-design and prospective evaluation of longitudinal supportive care approaches without establishing intervention effectiveness.

**Supplementary information:**

The online version contains supplementary material available at https://doi.org/10.1186/s12885-026-17245-y.

## Background

Chimeric antigen receptor T-cell therapy has improved outcomes for selected patients with relapsed or refractory hematologic malignancies, but treatment can involve substantial physical burden, intensive monitoring, and demanding transitions between inpatient and outpatient care. Clinician-reported toxicity does not capture all physical, emotional, social, and informational consequences experienced by patients. Patient-reported outcomes (PROs) therefore provide an important complement to clinical endpoints, yet remain incompletely integrated into routine CAR T-cell care and longitudinal research [1–4].

Prospective studies and reviews suggest that health-related quality of life (HRQoL) changes over the course of CAR T-cell therapy. Fatigue, physical functioning, and overall HRQoL may worsen during the acute treatment period and improve during recovery among survivors [2–5]. The relevance of these outcomes is underscored by evidence from multiple myeloma showing that patients may view quality of life as comparable in importance to progression-free survival when weighing treatment outcomes [6].

Beyond physical symptoms, patients may face uncertainty about efficacy and toxicity, fear of progression, social isolation, disruption of everyday life, and changing dependence on relatives. Qualitative studies have described communication gaps, family burden, and supportive care needs that may be missed by routine toxicity grading or very brief distress measures [7, 8]. Neuropsychiatric complications can add further complexity to the treatment course [9].

Digitally enabled supportive care may help extend symptom monitoring, psycho-oncological contact, education, and self-management support beyond hospitalization. In broader oncology populations, electronic patient-reported outcome monitoring has improved symptom detection and patient-reported outcomes [10, 11]. At the same time, unmet psychosocial and supportive care needs remain common across cancer and hematologic populations [12–14], suggesting that digital approaches are most useful when embedded within accessible multidisciplinary care.

Most CAR T-cell studies have examined standardized PRO trajectories, supportive care preferences, or qualitative experiences separately. This makes it difficult to see how measured changes in burden relate to patients’ own priorities within the same treatment course. We therefore examined longitudinal quality of life, psychological distress, loneliness, fear of progression, somatic symptom burden, perceived social support, structured supportive care preferences, and open-ended accounts across four phases before and after CAR T-cell therapy.

## Methods

### Study design, setting, and assessment schedule

This prospective single-center observational study was conducted within the CAR T-cell program of a German tertiary university hospital and is reported in accordance with the Strengthening the Reporting of Observational Studies in Epidemiology (STROBE) recommendations [15]. Assessments were scheduled by clinical phase. T0 was completed before CAR T-cell treatment; the exact interval between T0 and infusion was not captured as a standardized variable. T1 was completed during the index hospitalization after infusion and before discharge; the exact post-infusion day was likewise not standardized. T2 was completed on the day of discharge or close to discharge, and T3 seven to eight weeks after discharge. Length of stay and exact infusion-to-assessment intervals were not systematically recorded.

### Routine clinical care and treatment context

Treatment decisions were independent of study participation. Patients had already been approved for CAR T-cell therapy by the specialized hematology/cellular therapy team according to product-specific indications or clinical-trial protocols applicable at the time. Routine care generally included treatment counseling, leukapheresis, bridging therapy when clinically indicated, lymphodepleting conditioning, inpatient CAR T-cell infusion and monitoring for acute toxicities, discharge education, and specialized outpatient follow-up. Psycho-oncological consultation and other supportive services, including social counseling, physiotherapy or rehabilitation planning, and nutritional counseling, were available through routine referral according to clinical need and local availability. Relatives were involved where appropriate. The study did not alter clinical care, and individual use or intensity of supportive services was not systematically recorded.

### Participants and longitudinal analytic cohort

Adult patients with a hematologic malignancy scheduled to undergo CAR T-cell therapy at the study center were eligible. Inclusion criteria were age 18 years or older, planned CAR T-cell therapy, sufficient ability to complete German-language questionnaires, and written informed consent. Patients were excluded when participation was not feasible because of severe cognitive impairment, insufficient German-language comprehension, a critical clinical condition that precluded questionnaire completion, or withdrawal of consent.

**Fig. 1:**
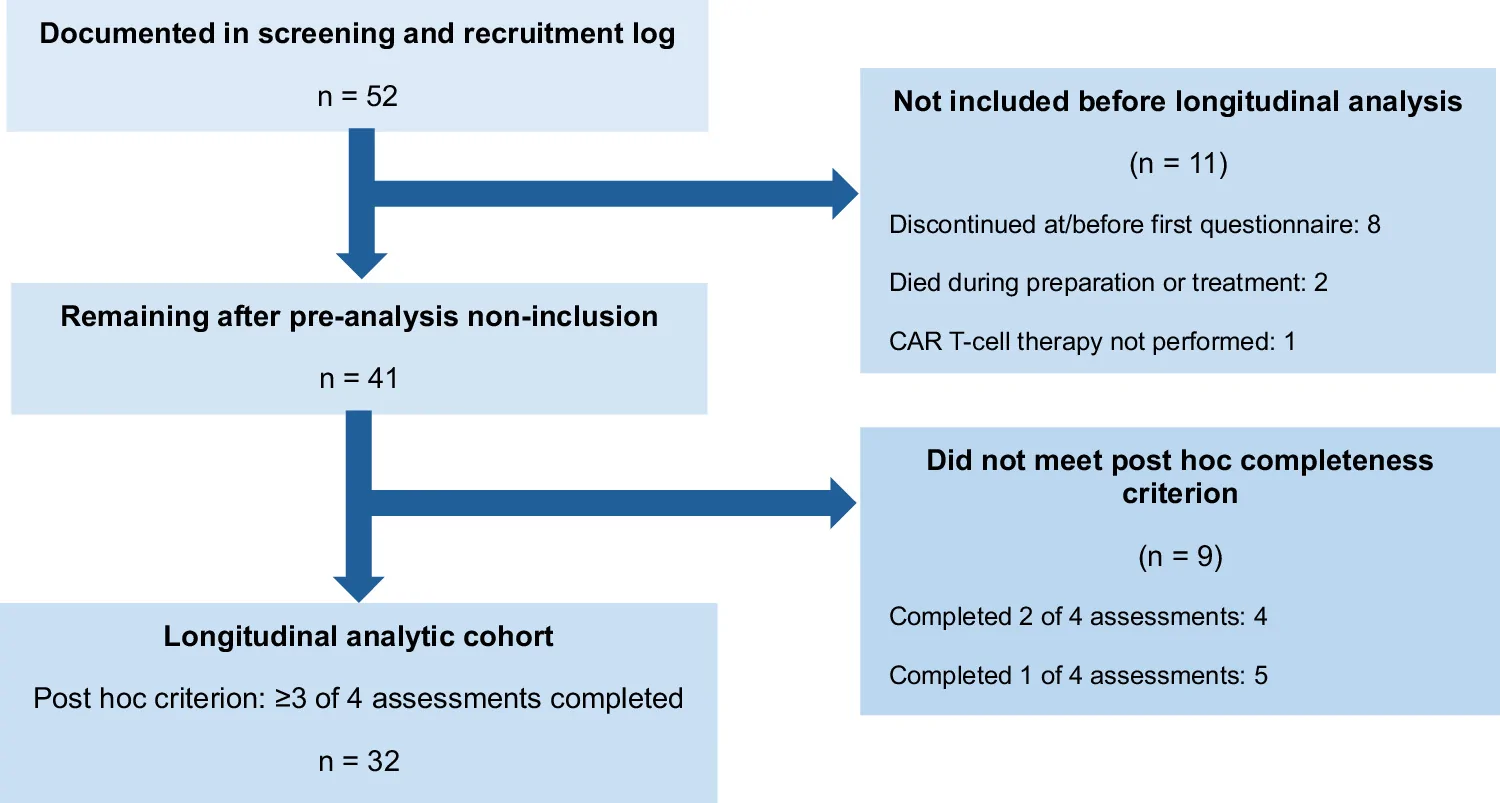
Participant flow to the longitudinal analytic cohort. The post hoc completeness criterion required at least three of four scheduled assessments and was applied after data collection for the present trajectory analysis. Outcome-specific missing values within included participants were retained in the generalized estimating equation models

Patients considered for study participation during the recruitment period were invited and documented in a screening/recruitment log. For the present longitudinal trajectory analysis, a post hoc completeness criterion required at least three of the four scheduled assessments. This criterion was applied after data collection and was not an eligibility criterion for study participation or treatment. It was chosen so that each participant in the analytic cohort contributed data from at least three phases of the treatment course. Participants with only one or two completed assessments were therefore not included in this analytic cohort. Within the cohort, all available scale-specific observations were retained. Numerical participant flow is reported in the Results and Fig. 1.

To contextualize the analytic cohort, available clinical variables were summarized descriptively: Eastern Cooperative Oncology Group (ECOG) performance status, number of prior therapy lines, CAR T-cell product, documented comorbidity, and maximum documented cytokine release syndrome (CRS) grade. These variables were not included as covariates in the longitudinal models because of the small sample and the exploratory study design. Detailed bridging and conditioning regimens, treatment toxicities beyond CRS, length of stay, and exact assessment dates were not available in a sufficiently complete and standardized form for analysis.

### Measures

Health-related quality of life was assessed using the European Organisation for Research and Treatment of Cancer Quality of Life Questionnaire-Core 30 (EORTC QLQ-C30) [16]; the QLQ-C30 summary score was calculated using the published higher-order scoring approach [17]. Anxiety and depressive symptoms were screened with the Patient Health Questionnaire-4 (PHQ-4) [18], loneliness with the 3-item UCLA Loneliness Scale [19], fear of progression with the short Fear of Progression Questionnaire (PA-F-KF/FoP-Q-SF) [20, 21], and somatic symptom burden with the Somatic Symptom Scale-8 (SSS-8) [22]. Perceived illness-related social support was assessed with the 8-item Illness-Specific Social Support Scale (SSUK-8/ISSS-8), including positive support and detrimental interaction subscales [23–25].

Participants also completed a clinician-developed, study-specific needs assessment addressing supportive care preferences and informational needs that were not fully covered by the standardized measures. The assessment was developed by an interdisciplinary team of psycho-oncologists and hematologists involved in routine CAR T-cell care. Item selection drew on clinical experience, published literature on supportive care needs in oncology, and recurring issues raised during psycho-oncological consultations with patients receiving intensive hematologic cancer treatment. The instrument was used for descriptive needs mapping and hypothesis generation and was not developed as a psychometrically validated scale.

The open-ended component covered four a priori areas: illness- and treatment-related concerns and determinants of quality of life; preferences for psycho-oncological, family, peer, and digital support; appraisal of the survey and support process; and recall or understanding of leukapheresis, bridging therapy, and conditioning therapy. This breadth reflected the exploratory aim of identifying intervention-relevant priorities across the treatment course. Because the questions were optional and questionnaire-based, no probing was possible and responses were often brief and variable. We therefore did not derive a formal knowledge score or interpret theme frequencies as prevalence estimates. The English-language instrument is provided as Additional file 1.

### Scoring

PHQ-4, UCLA-3, PA-F-KF, SSS-8, and SSUK-8 scores were calculated as summed scores when the required item information was available. EORTC QLQ-C30 scores were linearly transformed to a 0-100 scale according to the EORTC scoring manual. For functioning scales and global health status/quality of life, higher scores indicate better functioning or quality of life; for symptom scales, higher scores indicate greater symptom burden. The EORTC QLQ-C30 summary score was calculated according to the published scoring approach, excluding global health status/quality of life and financial difficulties [17].

Study-specific structured items were summarized as frequencies and percentages using the number of available responses for each item and timepoint as the denominator. Self-rated distress was assessed on a 0-10 numeric rating scale, with higher values indicating greater distress.

### Statistical analysis

Given the exploratory observational design and the limited number of patients treated at a single center, no formal sample size calculation was performed. Descriptive statistics are reported as mean ± standard deviation, median with interquartile range, or number with percentage, as appropriate. Longitudinal change was examined using generalized estimating equations with a Gaussian distribution, identity link, exchangeable working correlation structure, and time as the sole categorical predictor, with T0 as the reference. Regression coefficients therefore represent unadjusted mean differences from baseline. This approach used all available repeated outcome measurements within the analytic cohort despite outcome-specific missingness. No imputation was performed. Because multiple outcomes and contrasts were examined without multiplicity adjustment, p values are interpreted as nominal and hypothesis-generating.

### Thematic analysis of open-ended questionnaire responses

Open-ended responses from all available timepoints were analyzed using a descriptive thematic analysis informed by Braun and Clarke’s approach [26, 27]. We used a primarily inductive, semantic orientation. Given the questionnaire-based data and generally brief responses, the aim was to provide a structured account of recurring patterns of meaning rather than the depth expected from an interview-based interpretive study. Reporting was informed by qualitative reporting standards where applicable [28].

AF and SAB repeatedly read the complete response corpus, generated initial codes, grouped related codes into candidate themes, and iteratively reviewed the themes against both the coded extracts and the full dataset. Themes were developed across the pooled T0-T3 corpus rather than through a formal timepoint-specific qualitative comparison. Candidate themes were discussed with the multidisciplinary research team to assess clinical plausibility and consider alternative interpretations. Formal inter-rater reliability was not calculated because coding and theme development were treated as an iterative interpretive process. Saturation was not claimed.

AF is a physician and clinician scientist in psychosomatic medicine and psycho-oncology who coordinated the study; SAB is a doctoral researcher who contributed to data acquisition and qualitative analysis. Their clinical familiarity supported contextual interpretation and may also have shaped attention toward supportive care concerns. Reflexive checks included retaining statements that did not fit dominant patterns, repeatedly comparing candidate themes with the complete response set, and discussing interpretations with colleagues from psychosomatic medicine and hematology.

Selected quotations were translated from German into English by the research team and checked against the original responses. Wording was edited only where needed for readability without changing meaning. Quote labels denote pseudonymized screening identifiers and timepoints; identifiers are therefore not consecutive within the analytic cohort. The qualitative findings were not subjected to inferential testing and are not presented as estimates of theme prevalence.

### Integration of study components

After the quantitative outcomes, structured needs items, and open-ended responses had been analyzed separately, the research team compared their direction, timing, and content to identify areas of convergence, complementarity, and divergence. The integrated interpretation is reported narratively and in a joint display (Table 5).

## Results

### Participant flow and baseline characteristics

Of 52 patients documented in the screening/recruitment log, 32 met the post hoc completeness criterion and were included in the longitudinal analytic cohort. Twenty patients were not included: eight discontinued participation at or before completion of the first questionnaire, two died during treatment preparation or the treatment course after expressing willingness to participate, and one did not proceed to CAR T-cell therapy. A further nine did not meet the post hoc completeness criterion; four completed two of four assessments and five completed one of four assessments. The final cohort therefore comprised participants with at least three of four scheduled assessments (Fig. 1). It included 17 male and 15 female participants, with a median age of 61.0 years (IQR 53.0-65.5; mean 58.3 ± 11.2 years). Multiple myeloma was the most common diagnosis (23/32, 71.9%). Among the 31 participants with recorded ECOG status, 30 had ECOG 0-1. The median number of prior therapy lines was 3 (range 1-7), 26/32 participants had at least one documented comorbidity, and ciltacabtagene autoleucel was the most common CAR T-cell product (20/32, 62.5%). Maximum documented CRS grade was 0 in 11 participants, 1 in 12, 2 in eight, and 3 in one. Baseline and treatment-related characteristics are summarized in Table 1.

**Table 1:** Baseline and treatment-related characteristics of the study cohort

Characteristic	Value
Patients included in longitudinal analysis, n	32
Age, mean ± SD	58.3 ± 11.2 years
Age, median (IQR)	61.0 (53.0-65.5) years
Sex, male/female, n (%)	17 (53.1) / 15 (46.9)
Stable partnership/married, n (%)	25 (78.1)
Multiple myeloma, n (%)	23 (71.9)
Diffuse large B-cell lymphoma, n (%)	4 (12.5)
Other hematologic malignancies, n (%)	5 (15.6)
ECOG performance status 0/1/2/not recorded, n (%)	12 (37.5) / 18 (56.3) / 1 (3.1) / 1 (3.1)
Prior therapy lines, median (range)	3 (1-7)
Any documented comorbidity, n (%)	26 (81.3)
Cardiovascular comorbidity, n (%)	16 (50.0)
CAR T-cell product, n (%)	Ciltacabtagene autoleucel 20 (62.5); axicabtagene ciloleucel 7 (21.9); idecabtagene vicleucel 3 (9.4); brexucabtagene autoleucel 1 (3.1); lisocabtagene maraleucel 1 (3.1)
Maximum documented CRS grade 0/1/2/3, n (%)	11 (34.4) / 12 (37.5) / 8 (25.0) / 1 (3.1)

*ECOG* eastern cooperative oncology group, *CRS* cytokine release syndrome

Other hematologic malignancies comprised primary mediastinal large B-cell lymphoma, transformed follicular lymphoma, Hodgkin lymphoma, acute lymphoblastic leukemia, and chronic lymphocytic leukemia (n = 1 each). Comorbidity categories may overlap. Clinical variables are presented descriptively and were not entered into the longitudinal models

### Longitudinal descriptive findings

Scale-specific sample sizes varied across outcomes and timepoints and are reported in Table 2. The least favorable mean values for global health status/quality of life, physical functioning, fatigue, somatic symptom burden, and the EORTC QLQ-C30 summary score occurred at T2, around discharge. By T3, seven to eight weeks after discharge, most EORTC domains had returned to baseline levels or had improved descriptively. PHQ-4 scores changed little across timepoints, fear-of-progression scores were descriptively lower after baseline, and loneliness increased transiently during the inpatient phase.

**Table 2:** Longitudinal descriptive scores across study timepoints

Outcome	T0	T1	T2	T3
**EORTC global health status/QoL**	55.73 ± 25.53 (n = 32)	52.38 ± 26.82 (n = 28)	44.55 ± 24.26 (n = 26)	64.58 ± 22.20 (n = 32)
**EORTC physical functioning**	74.62 ± 27.83 (n = 31)	71.43 ± 27.33 (n = 28)	61.11 ± 30.94 (n = 24)	74.67 ± 24.28 (n = 30)
**EORTC emotional functioning**	66.67 ± 27.68 (n = 32)	68.15 ± 27.03 (n = 28)	64.42 ± 29.30 (n = 26)	70.97 ± 24.80 (n = 31)
**EORTC social functioning**	57.29 ± 36.89 (n = 32)	54.17 ± 40.98 (n = 28)	54.67 ± 38.04 (n = 25)	61.98 ± 29.09 (n = 32)
**EORTC fatigue**	40.62 ± 34.64 (n = 32)	50.79 ± 34.00 (n = 28)	58.12 ± 38.91 (n = 26)	41.94 ± 31.13 (n = 31)
**EORTC QLQ-C30 summary score**	72.28 ± 23.13 (n = 30)	66.33 ± 24.64 (n = 27)	64.74 ± 25.70 (n = 21)	75.34 ± 18.82 (n = 28)
**PHQ-4 total**	2.29 ± 2.64 (n = 31)	2.57 ± 2.79 (n = 27)	2.65 ± 3.15 (n = 26)	1.84 ± 1.82 (n = 32)
**UCLA-3 loneliness**	2.16 ± 2.75 (n = 32)	2.96 ± 3.10 (n = 28)	2.38 ± 2.90 (n = 26)	2.62 ± 3.05 (n = 32)
**Fear of progression (PA-F-KF)**	16.60 ± 10.36 (n = 30)	13.32 ± 9.75 (n = 25)	13.86 ± 9.10 (n = 21)	13.19 ± 8.52 (n = 27)
**SSS-8 somatic symptom burden**	6.71 ± 5.34 (n = 31)	7.30 ± 6.26 (n = 28)	8.73 ± 7.33 (n = 26)	6.81 ± 5.28 (n = 32)
**SSUK-8 positive support**	13.59 ± 2.82 (n = 32)	13.61 ± 2.88 (n = 28)	14.58 ± 1.72 (n = 26)	13.34 ± 2.66 (n = 32)
**SSUK-8 detrimental interactions**	2.74 ± 2.27 (n = 31)	2.54 ± 2.22 (n = 28)	3.23 ± 2.86 (n = 26)	2.62 ± 2.35 (n = 32)

Values are mean ± SD; n denotes available observations. T0, before CAR T-cell treatment (exact interval to infusion not standardized); T1, during the post-infusion index hospitalization and before discharge (exact day not standardized); T2, at or around hospital discharge; T3, seven to eight weeks after discharge

### Longitudinal models

Exploratory unadjusted generalized estimating equation analyses are summarized in Table 3. Because no multiplicity adjustment was applied, all p values and individual contrasts are interpreted as nominal.

**Table 3:** Generalized estimating equation models with T0 as reference

Outcome	Overall p	T1 vs T0 β	T1 p	T2 vs T0 β	T2 p	T3 vs T0 β	T3 p
**PHQ-4 total**	0.205	0.329	0.336	0.200	0.682	-0.507	0.142
**UCLA-3 loneliness**	0.004	0.795	0.009	0.125	0.502	0.469	0.149
**Fear of progression (PA-F-KF)**	0.078	-2.389	0.032	-2.317	0.041	-2.766	0.033
**SSS-8 somatic symptom burden**	0.379	0.983	0.211	1.925	0.093	0.160	0.748
**SSUK-8 positive support**	0.033	-0.138	0.785	0.570	0.114	-0.250	0.371
**SSUK-8 detrimental interactions**	0.677	-0.047	0.918	0.346	0.585	-0.187	0.699
**EORTC global health status/QoL**	0.001	-4.737	0.349	-10.026	0.066	8.854	0.051
**EORTC physical functioning**	0.162	-4.973	0.225	-14.450	0.046	-0.813	0.825
**EORTC emotional functioning**	0.712	-0.799	0.786	-0.295	0.964	3.534	0.345
**EORTC social functioning**	0.525	-3.900	0.545	-2.132	0.696	4.688	0.379
**EORTC fatigue**	0.002	11.990	0.015	15.574	0.008	0.851	0.859
**EORTC QLQ-C30 summary score**	0.006	-7.772	0.006	-6.954	0.035	0.752	0.745

β values are unadjusted mean differences relative to T0 from Gaussian generalized estimating equation models with an exchangeable working correlation structure. Overall p values test the effect of time. Timepoint definitions are provided in the Table 2 note

**Fig. 2:**
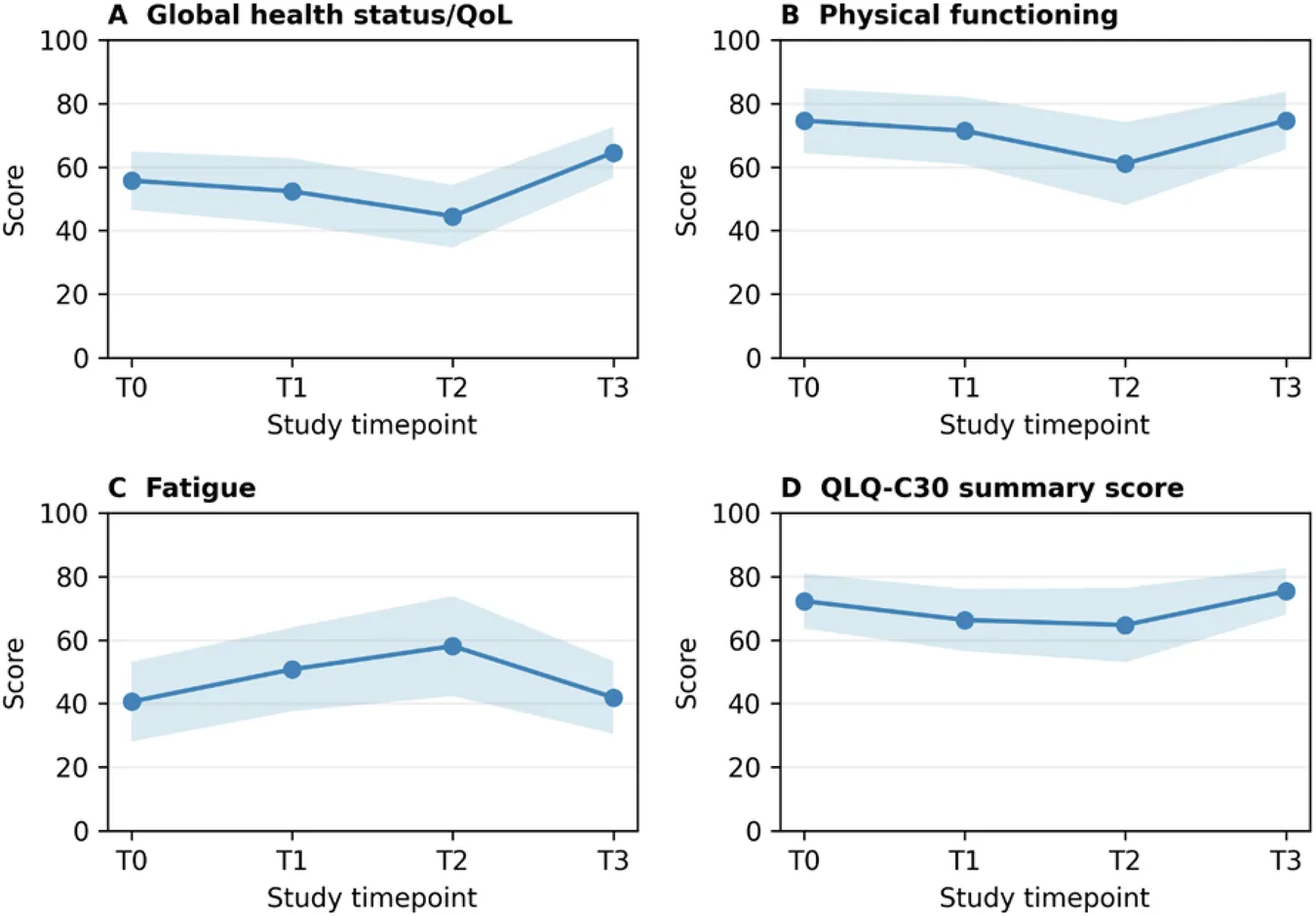
Longitudinal trajectories of key EORTC domains. Observed means with pointwise 95% confidence intervals based on available observations are shown for (**A**) global health status/quality of life, (**B**) physical functioning, (**C**) fatigue, and (**D**) the EORTC QLQ-C30 summary score across T0-T3. Higher scores indicate better status for global health status/quality of life, physical functioning, and the summary score, whereas higher fatigue scores indicate greater symptom burden. The figure is descriptive and does not account for within-participant correlation; longitudinal model results are presented in Table 3. T3 was seven to eight weeks after discharge

Global health status/quality of life showed an overall time effect (p = 0.001), with a descriptive decline to T2 and recovery by T3; none of the individual contrasts reached the nominal 0.05 threshold. Physical functioning showed no overall time effect (p = 0.162), although the T2 contrast was nominally lower than baseline (β = -14.450, p = 0.046). Fatigue showed an overall time effect (p = 0.002) and was nominally higher at T1 (β = 11.990, p = 0.015) and T2 (β = 15.574, p = 0.008). The EORTC QLQ-C30 summary score also showed an overall time effect (p = 0.006) and was nominally lower at T1 (β = -7.772, p = 0.006) and T2 (β = -6.954, p = 0.035). These trajectories are shown in Fig. 2.

**Fig. 3:**
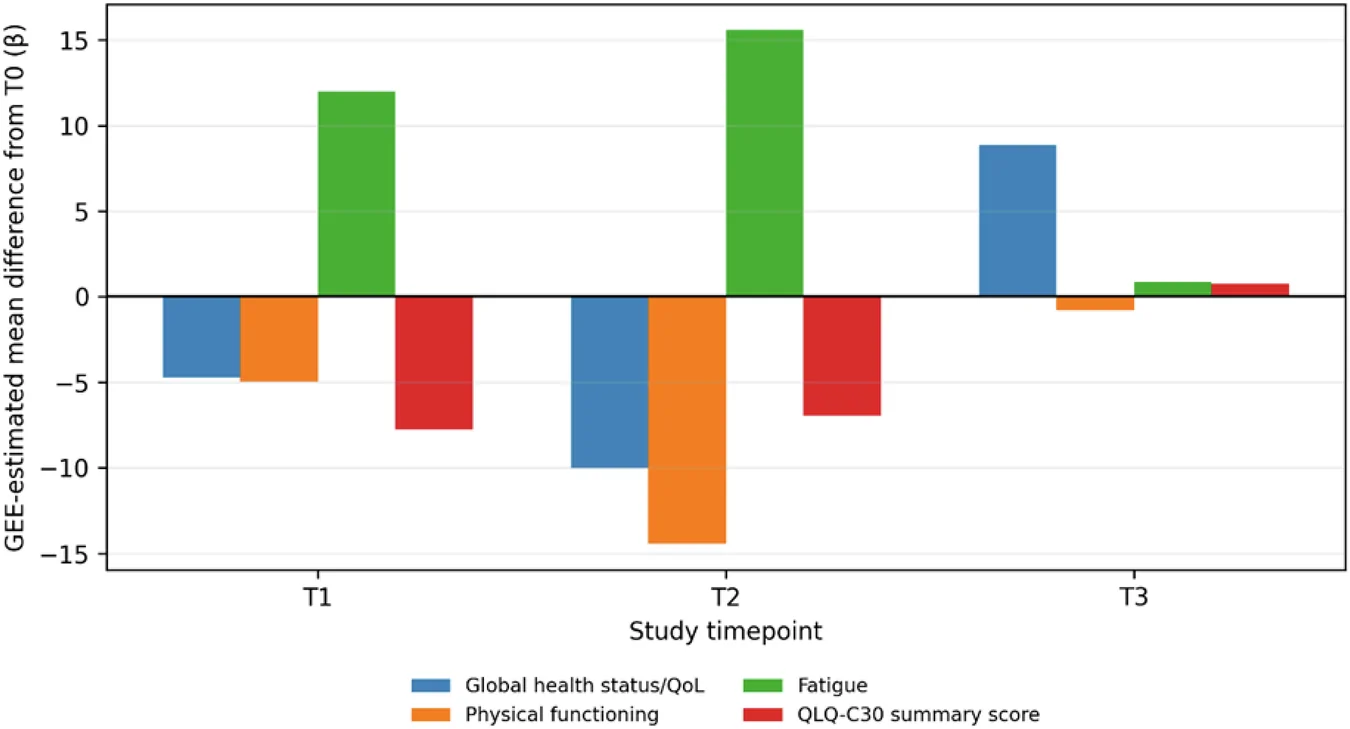
GEE-estimated mean differences from T0 across study timepoints. The bars show unadjusted mean differences from T0 (β) estimated by the generalized estimating equation models for global health status/quality of life, physical functioning, fatigue, and the EORTC QLQ-C30 summary score at T1, T2, and T3. For global health status/quality of life, physical functioning, and the summary score, negative β values indicate poorer outcomes than at T0; for fatigue, positive β values indicate greater symptom burden. Corresponding p values and overall time effects are reported in Table 3. T3 was seven to eight weeks after discharge

PHQ-4 scores changed little over time and showed no overall time effect. Loneliness showed an overall time effect (p = 0.004) and was nominally higher at T1 than at baseline (β = 0.795, p = 0.009), but not at T2 or T3. Fear-of-progression scores were descriptively lower after baseline; although the individual contrasts were nominally below 0.05, the overall time effect did not reach that threshold (p = 0.078), so this pattern should be interpreted cautiously. Positive social support showed an overall time effect (p = 0.033), but no individual timepoint differed nominally from baseline. Somatic symptom burden and detrimental interactions showed no overall time effects. Unadjusted GEE estimates for selected EORTC outcomes are shown in Fig. 3.

### Structured supportive care preferences

Across the study period, substantial proportions of participants considered psycho-oncological, family-oriented, peer-based, and digital support useful. Post-discharge psycho-oncological or psychotherapeutic support was endorsed by 60.0% to 70.0% of respondents, and support offers for relatives by 52.0% to 74.1%. Long waiting times for psycho-oncological care were considered burdensome by 53.3% to 64.5%. Interest in an online stress-reduction intervention ranged from 46.4% to 60.0%, while 66.7% to 79.3% considered online contact with patients who had already undergone CAR T-cell therapy useful. Mean self-rated distress was highest at T1 during hospitalization and decreased to 3.88 at T3. These descriptive findings reflect preferences and perceived usefulness; they do not measure actual service use or intervention effectiveness (Table 4).

**Table 4:** Selected supportive care preferences and self-rated distress over time

Preference/indicator	T0	T1	T2	T3
Support after discharge considered useful	18/29 (62.1%)	17/28 (60.7%)	15/25 (60.0%)	21/30 (70.0%)
Support offers for relatives considered useful	20/27 (74.1%)	17/27 (63.0%)	13/25 (52.0%)	17/31 (54.8%)
Long waiting times for psycho-oncology considered burdensome	16/30 (53.3%)	17/27 (63.0%)	15/26 (57.7%)	20/31 (64.5%)
Interest in online stress-reduction intervention	16/29 (55.2%)	13/28 (46.4%)	15/25 (60.0%)	17/31 (54.8%)
Online contact with patients who had already undergone CAR T-cell therapy considered useful	22/29 (75.9%)	23/29 (79.3%)	18/27 (66.7%)	21/30 (70.0%)
Mean self-rated distress (0-10)	5.25 ± 3.48	6.21 ± 2.85	5.89 ± 2.90	3.88 ± 2.79

Values are n/N (%) unless otherwise stated. Percentages use available item responses at each timepoint as the denominator. Timepoint definitions are provided in the Table 2 note

**Fig. 4:**
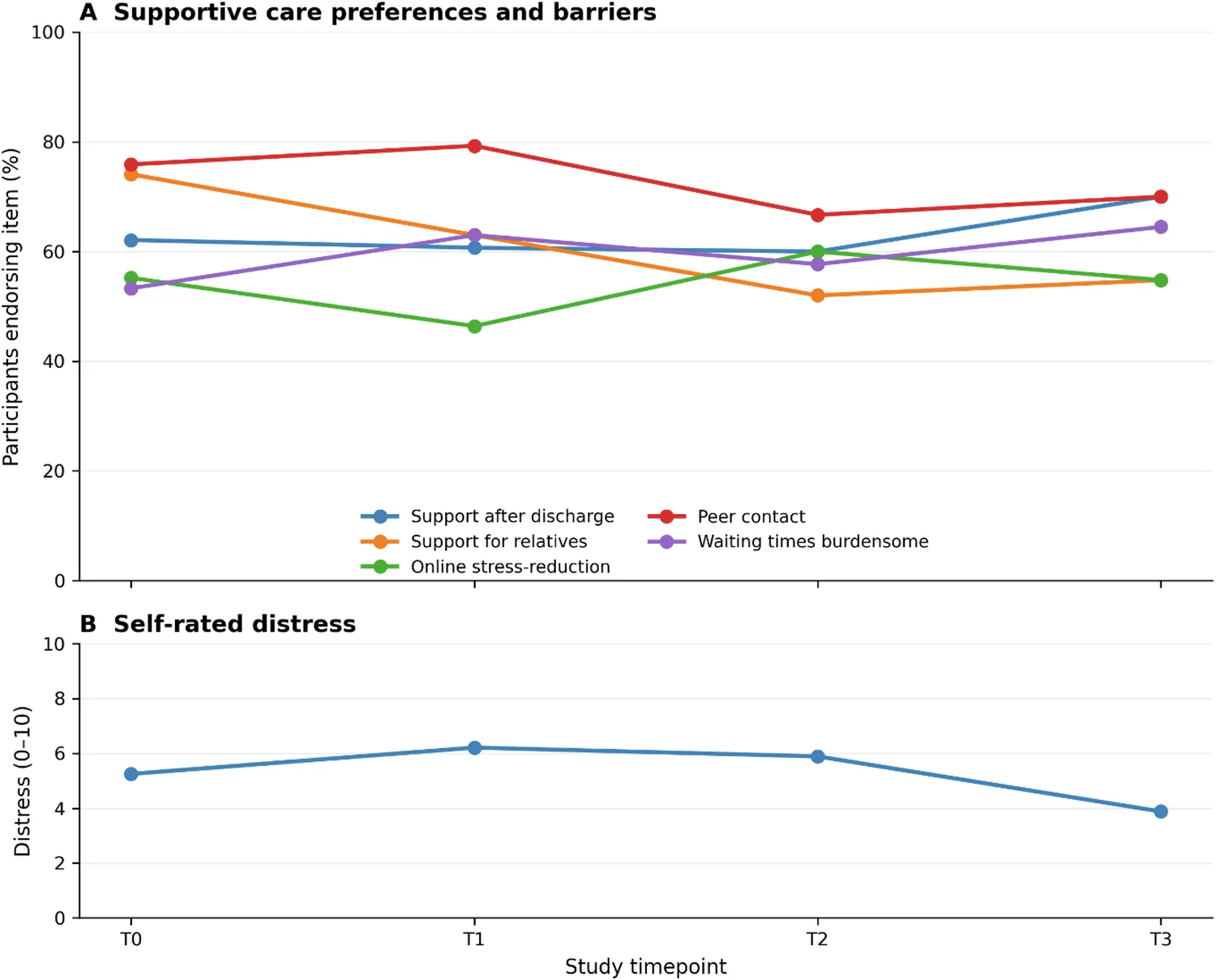
Supportive care preferences, barriers, and distress over time. Panel (**A**) shows the proportion of participants endorsing psycho-oncological support after discharge, support for relatives, online stress reduction, peer contact, and the perception that waiting times for psycho-oncological care are burdensome. Percentages are based on available item responses, and denominators vary by item and timepoint; no inferential testing was performed for these study-specific items. Panel (**B**) shows mean self-rated distress (0-10). T3 was seven to eight weeks after discharge

The longitudinal patterns of supportive care preferences, perceived barriers, and self-rated distress are shown in Fig. 4.

**Fig. 5:**
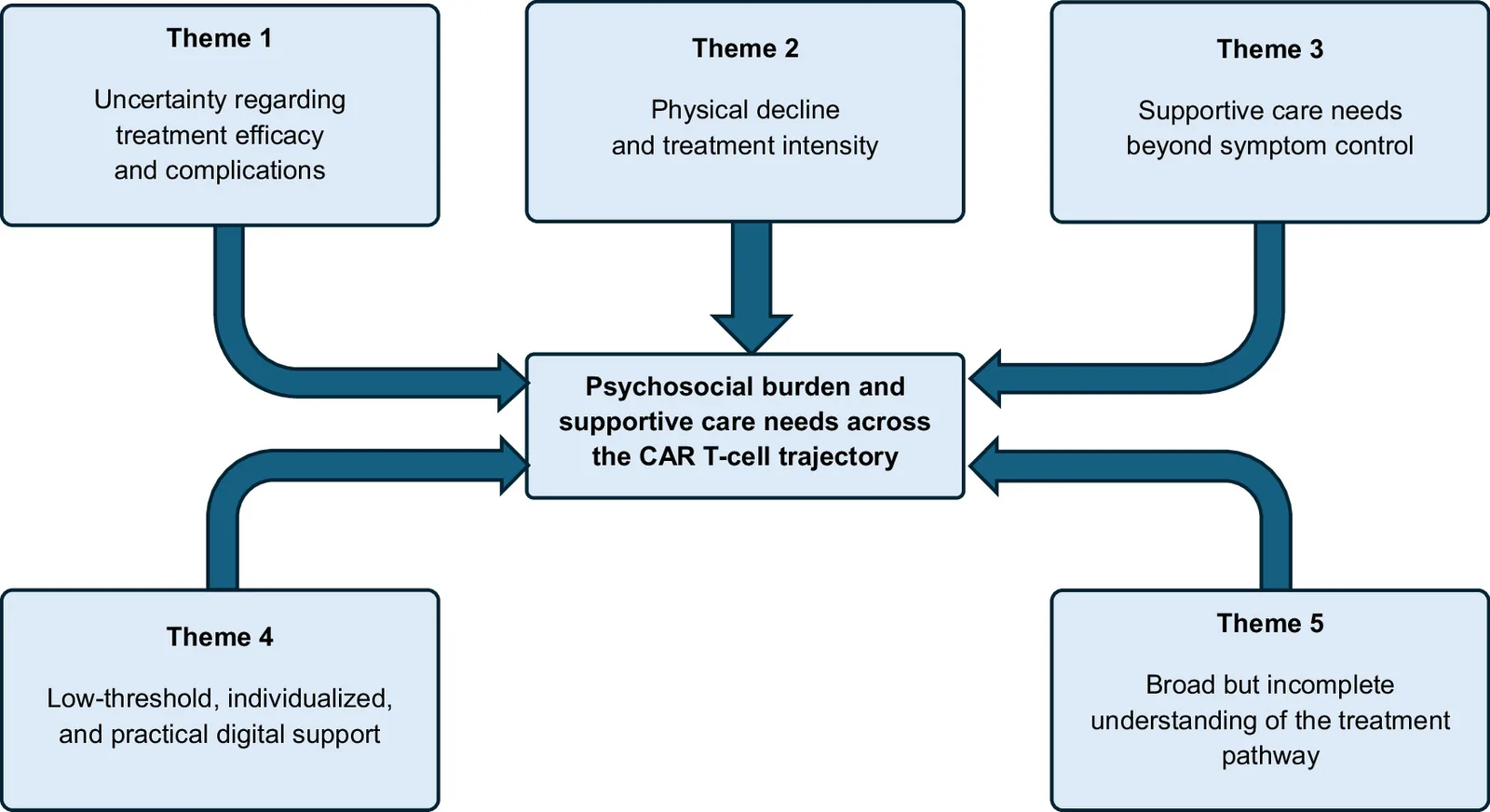
Thematic map of open-ended responses. The five themes summarize recurring patterns across all available T0-T3 free-text responses. The map is descriptive and does not indicate frequency, prevalence, or causal relationships

Open-ended responses added context to the standardized results by showing how patients described their concerns, support priorities, and expectations regarding digital and relational support. The thematic structure is summarized in Fig. 5.

### Qualitative findings from open-ended responses

The descriptive thematic analysis generated five themes: (1) living with uncertainty regarding treatment efficacy and complications, (2) physical decline and treatment intensity as major determinants of burden, (3) supportive care needs extending beyond symptom control, (4) preferences for low-threshold, individualized, and practical digital support, and (5) broad but incomplete understanding of the treatment pathway. Selected translated quotations illustrate each theme.

### Theme 1: Living with uncertainty regarding treatment efficacy and complications

Across timepoints, patients repeatedly described uncertainty about whether CAR T-cell therapy would work and whether remission could be achieved or maintained. Concerns about treatment failure, relapse, and an unpredictable disease course appeared throughout the study period. Side effects and treatment-related complications were also mentioned, especially around treatment initiation and early follow-up. Neurotoxicity was a specific concern for some participants, while others explicitly reported no major concerns, showing marked variation in how the treatment course was experienced.

Illustrative quotes included: “What happens next if this therapy does not help?” (P12, T0), and “I worry that the result will not be as hoped; the doctor said a few years of peace, and I am afraid of disappointment.” (P37, T3). In contrast, another participant stated: “I have no major concern in this regard.” (P13, T0).

### Theme 2: Physical decline and treatment intensity as major determinants of burden

Patients described quality of life as strongly influenced by physical symptoms and treatment intensity. Frequently mentioned burdens included fatigue, pain, weakness, reduced mobility, impaired general condition, hospitalization, and the cumulative demands of ongoing treatment. These accounts were consistent with the quantitative pattern in fatigue and physical functioning. Participants also linked poorer quality of life to dependence on others, uncertainty about recovery, and disruption of everyday life.

Illustrative quotes reflected both acute and later functional burden: “Tiredness, weakness, reduced general condition, hardly any holidays possible, and rehabilitation postponed” (P6, T0), and “Fatigue, concentration problems, weakness, slowness, and reduced endurance” (P28, T3).

### Theme 3: Supportive care needs extending beyond symptom control alone

Open-ended responses showed that patients viewed support as broader than symptom control. Alongside psycho-oncological care, they mentioned support for relatives, exercise and rehabilitation, nutritional guidance, clear contact structures, self-help groups, and opportunities for peer exchange. The responses therefore pointed to practical, relational, and functional support needs as well as emotional support.

Participants described supportive care needs as practical, relational, and family-oriented. Illustrative quotes included: “More information offers for relatives, educational brochures” (P4, T0), “Contact with other patients in the same situation as me” (P14, T0), and “Education at discharge after CAR T-cell therapy about behavior at home, side effects and support options, duration of follow-up treatment, recovery time, and rehabilitation” (P4, T3).

### Theme 4: Preferences for low-threshold, individualized, and practically useful digital support

Patients described specific expectations for digital psycho-oncological or supportive care. They valued straightforward access, ease of use, understandable content, flexible availability, and adaptation to individual concerns. Practical coping strategies, concise formats, and access to professional guidance were also important. These responses suggest that acceptability depends on how well a digital offer fits patients’ needs and treatment context, as well as on its availability.

Illustrative quotes emphasized usability and individual relevance: “Keep it simple, with short and understandable texts, and avoid redundancies” (P10, T0), “It should address my own problems and concerns” (P21, T0), and “Uncomplicated internet access with a password, professional guidance, and a doctor in the background who provides medically correct information; just knowing that I could talk to someone online whenever I feel bad would be relieving” (P25, T3).

### Theme 5: Broad but incomplete understanding of the treatment pathway

Responses to the open-ended knowledge questions suggested that many participants retained a broad understanding of the CAR T-cell pathway. They commonly referred to leukapheresis or cell collection, bridging treatment, chemotherapy, cell infusion, and possible side effects. Leukapheresis was usually understood as collection or separation of leukocytes or T cells; bridging therapy as interim treatment until CAR T-cell infusion; and conditioning therapy as chemotherapy before infusion. Partial understanding, uncertainty, and non-response were nevertheless present across all three concepts.

Examples of retained procedural knowledge included: “T cells are collected from the blood, modified in the laboratory, and then returned to the body to detect and fight cancer cells” (P22, T0), and “Bridging therapy is treatment until the CAR T cells are administered” (P3, T3). At the same time, incomplete understanding remained visible, for example when participants answered “not known to me” regarding conditioning chemotherapy (P8, T0) or left items unanswered.

### Integration of longitudinal outcomes, supportive care preferences, and open-ended findings

Viewed together, the three study components identified hospitalization and discharge as the period of greatest vulnerability and showed that brief distress screening did not capture all patient-identified needs. Less favorable physical EORTC scores were consistent with written descriptions of fatigue, weakness, impaired mobility, and dependence. The transient rise in loneliness occurred alongside sustained interest in peer contact and support for relatives. PHQ-4 scores changed little, yet participants continued to describe uncertainty about efficacy, complications, and relapse and to endorse psycho-oncological support. The digital and knowledge findings added practical detail about how future support might be delivered (Table 5).

**Table 5:** Joint display integrating study components

Domain	Evidence across study components	Integrated interpretation
**Physical and functional burden**	Longitudinal outcomes: physical functioning was lowest at T2; fatigue was higher and the EORTC summary score lower at T1/T2. Structured indicator: distress was highest at T1 and remained elevated at T2. Open-ended responses: fatigue, weakness, reduced mobility, and dependence.	The findings converge on hospitalization and discharge as key phases for functional and symptom-oriented support.
**Emotional and illness-related uncertainty**	Longitudinal outcomes: PHQ-4 remained stable; fear of progression showed no overall time effect. Structured preference: 60.0% to 70.0% endorsed post-discharge psycho-oncological support. Open-ended responses: concerns about efficacy, complications, relapse, and the future.	Brief anxiety/depression screening captured only part of the illness-related uncertainty and support needs described by participants.
**Social and caregiver needs**	Longitudinal outcome: loneliness increased transiently at T1. Structured preferences: 52.0% to 74.1% endorsed support for relatives and 66.7% to 79.3% peer contact. Open-ended responses: requests for family information and contact with patients in similar situations.	Caregiver information, social connection, and optional peer support emerged as related priorities.
**Digital and informational support**	Structured preference: 46.4% to 60.0% expressed interest in online stress reduction. Open-ended responses: simple, low-threshold, individualized, professionally guided tools; broad but incomplete understanding of the treatment pathway.	Participants favored concise, accessible, clinically connected digital support that reinforces education alongside personal care.

This joint display synthesizes separately analyzed components. It is interpretive and does not represent additional statistical testing

## Discussion

Across the three study components, hospitalization and discharge emerged as the period of greatest reported burden. EORTC scores were least favorable around discharge, and patients’ written responses described fatigue, weakness, reduced mobility, and dependence on others. By seven to eight weeks after discharge, most EORTC domains had returned to baseline levels or improved descriptively. PHQ-4 scores changed little, yet participants continued to describe uncertainty and to endorse psycho-oncological, family, and peer support. This combination of findings shows why brief anxiety/depression screening captured only part of the support priorities in this cohort.

The EORTC pattern is broadly consistent with prospective studies and reviews describing early deterioration followed by recovery among CAR T-cell survivors [1–5]. The lowest scores around discharge could reflect the cumulative burden of conditioning, infusion, acute monitoring, hospitalization, and deconditioning, although this dataset cannot attribute changes to specific clinical events. Improvement by T3 should also be interpreted in the context of an analytic cohort that remained able to complete repeated questionnaires. The emphasis on quality of life is especially relevant in a cohort dominated by multiple myeloma, where patients have rated quality of life as comparable in importance to progression-free survival [6].

PHQ-4 scores changed little across the four assessments. As an ultra-brief screen for anxiety and depressive symptoms, the PHQ-4 captures only part of the broader CAR T-cell experience. Participants still endorsed psycho-oncological support and described uncertainty about treatment success, complications, relapse, recovery, and future options. Similar concerns about communication and support have been reported in qualitative CAR T-cell research [7, 8], and unmet psychosocial needs remain common across oncology [12–14]. Brief symptom screening may therefore need to be complemented by questions about illness-related uncertainty, practical concerns, relationships, and support preferences.

Social needs were also apparent. Loneliness increased transiently during the inpatient phase, while interest in peer contact remained high and support for relatives was frequently considered useful. Hospitalization, protective precautions, distance from usual roles, and dependence on specialized care may contribute to social disconnection even when anxiety and depressive symptoms do not rise in parallel. Caregiver studies in CAR T-cell therapy likewise describe burden, distress, and prognostic uncertainty [29–31]. Our findings support further exploration of caregiver-inclusive information and optional peer exchange, while providing no evidence about the effectiveness of a particular format.

The fear-of-progression results also show the value of considering the quantitative and open-ended findings together. Mean scores were descriptively lower after baseline and several individual contrasts were nominally below 0.05, but the overall time effect was not. At the same time, participants repeatedly wrote about treatment failure, relapse, and an uncertain future. The difference may reflect the construct measured, assessment timing, response adaptation, limited power, or a combination of these factors. The open-ended findings therefore add context to a scale that did not show a robust overall longitudinal effect.

The digital findings went beyond a general preference for online care. Interest in online stress reduction was moderate, whereas interest in peer contact was consistently higher. Participants emphasized simple access, understandable and concise content, flexible availability, personalization, and a clear link to professional support. Evidence from broader oncology shows that electronic patient-reported outcome monitoring can improve symptom recognition and patient-reported outcomes [10, 11], but the present study did not test a digital intervention. Its contribution is to describe patient priorities that can be taken forward into co-design, feasibility testing, and later effectiveness evaluation.

Responses to the knowledge items also suggest that one-time education may be difficult to retain across a physically and cognitively demanding treatment course. Participants often remembered the broad purpose of leukapheresis, bridging therapy, and conditioning therapy, but understanding was not always complete. Repeated, phase-specific explanations, teach-back, and accessible written or digital reinforcement may therefore be worth evaluating around transitions in care. Any digital resource should remain connected to direct clinical communication and provide a clear route to professional advice.

These findings need to be read within the local care context. Participants were treated in a German tertiary CAR T-cell program with specialized hematologic follow-up and referral-based supportive services. Treatment eligibility had already been determined according to the product-specific indications or protocols applicable at the center. Existing counseling and support may have influenced both reported burden and preferences, although individual use of supportive services was not recorded. Transferability may therefore differ in settings with other access criteria, inpatient pathways, caregiver requirements, or supportive care resources.

Several limitations relate to sampling and clinical characterization. This was a single-center study with a modest sample, and 32 of 52 patients in the screening/recruitment log entered the longitudinal analytic cohort. Requiring at least three of four assessments helped ensure that included participants contributed data across several phases, but the criterion was applied post hoc and may have selected patients who survived, remained clinically stable, and were able or willing to complete repeated questionnaires. Selection bias related to questionnaire completeness therefore cannot be excluded, and scale-specific missingness remained within the cohort. The sample was dominated by multiple myeloma (71.9%) and ciltacabtagene autoleucel (62.5%); 30/31 participants with recorded ECOG status had ECOG 0-1. Transferability to other disease groups, products, and patients with poorer performance status is consequently limited. Performance status, prior therapy lines, comorbidity, CAR T-cell product, and CRS could be described but not incorporated into adjusted longitudinal analyses. Detailed bridging and conditioning regimens, length of stay, exact assessment days, and toxicities beyond CRS were not captured consistently, leaving residual clinical heterogeneity and confounding.

Measurement and interpretation also have limitations. Multiple outcomes and contrasts were tested without adjustment, so all p values are nominal and the risk of type I error is increased. The study-specific needs assessment was not a validated scale; endorsement reflects perceived usefulness rather than actual unmet need, uptake, or efficacy. The open-ended prompts intentionally covered broad domains and were optional, with no opportunity for probing. Responses were therefore brief and heterogeneous, saturation was not claimed, and the themes should not be read as prevalence estimates. The analysts’ clinical backgrounds may also have influenced interpretation despite reflexive checks and multidisciplinary discussion. Finally, T3 occurred seven to eight weeks after discharge, so longer-term trajectories remain unknown.

Strengths include prospective assessment across four clinically meaningful phases, simultaneous measurement of several psychosocial and quality-of-life domains, direct elicitation of supportive care and digital preferences, transparent participant flow, expanded clinical characterization, and explicit integration of quantitative and open-ended findings. Future multicenter studies can build on these observations by including richer treatment characterization, preplanned handling of missing data, longer follow-up, and prospective co-design and evaluation of supportive care approaches.

## Conclusion

In this single-center cohort, patient-reported physical and functional burden was greatest during hospitalization and around discharge and improved by seven to eight weeks after discharge. Brief anxiety/depression scores changed little, while participants continued to describe treatment-related uncertainty and to value psycho-oncological, caregiver, peer, informational, and individualized digital support. These findings can inform the co-design and prospective evaluation of longitudinal supportive care approaches around CAR T-cell therapy; they do not establish the effectiveness of any specific intervention.

## Supplementary information

Supplementary Information

## Data Availability

Deidentified participant data and analysis code generated and analyzed during the current study are available from the corresponding author upon reasonable request, subject to institutional, ethical, and legal restrictions.

## Abbreviations

CAR T-cell: chimeric antigen receptor T-cell
CRS: cytokine release syndrome
EORTC QLQ-C30: European Organisation for Research and Treatment of Cancer Quality of Life Questionnaire-Core 30
ePRO: electronic patient-reported outcome
GEE: generalized estimating equation
HRQoL: health-related quality of life
PA-F-KF: short Fear of Progression Questionnaire
PHQ-4: Patient Health Questionnaire-4
PRO: patient-reported outcome
QoL: quality of life
SSS-8: Somatic Symptom Scale-8
SSUK-8: 8-item Illness-Specific Social Support Scale
UCLA-3: 3-item UCLA Loneliness Scale
