# Supplementary material for: Longitudinal psychosocial burden, quality of life, and digital supportive care preferences before and after CAR T-cell therapy: a prospective single-center observational study
Source: BMC Cancer. 2026 Oct 27;26:1328. doi: 10.1186/s12885-026-17245-y (PMC13723932; doi:10.1186/s12885-026-17245-y)
Supplement: Supplementary file 1 [file 12885_2026_17245_MOESM1_ESM.docx]

**Additional file 1**

**English-language version of the clinician-developed, study-specific supportive care preferences and needs assessment**

This additional file provides the English translation of the clinician-developed, study-specific needs assessment used to assess supportive care preferences, informational needs, treatment understanding, and open-ended patient perspectives. The instrument was developed for this study and was used descriptively; it was not intended as a psychometrically validated scale. Standardized validated instruments administered elsewhere in the study, such as the EORTC QLQ-C30, PHQ-4, UCLA-3, PA-F-KF/FoP-Q-SF, SSS-8, and SSUK-8, are not reproduced here.

**Administration and timepoints**

| **Timepoint** | **Description** |
| --- | --- |
| T0 | Before CAR T-cell treatment. |
| T1 | During the index hospitalization after CAR T-cell infusion and before discharge. |
| T2 | On the day of hospital discharge or close to discharge after CAR T-cell therapy. |
| T3 | Approximately seven to eight weeks after hospital discharge. |

Items 1, 2, 4a, 5, 7, 9a, and 9b were baseline-only items. Diagnosis, relationship status, employment status, and items 10-44 were assessed longitudinally across study timepoints, with minor administrative numbering differences across the T1-T3 forms.

**A. Administrative, sociodemographic, and disease-related items**

| **No.** | **English item wording** | **Response format / options** | **Timepoints** |
| --- | --- | --- | --- |
| ID | Patient identification number | Administrative pseudonymized study ID; not a patient-facing question. | T0-T3 |
| 1 | How old are you? | Age in years. | T0 |
| 2 | What is your sex/gender? | Male; Female; Diverse. | T0 |
| 3 | For which diagnosis are you receiving CAR T-cell therapy? | Multiple myeloma; diffuse large B-cell lymphoma; follicular lymphoma; follicular lymphoma transformed to DLBCL; high-grade B-cell lymphoma; primary mediastinal large B-cell lymphoma; mantle cell lymphoma; marginal zone lymphoma; Hodgkin lymphoma; acute lymphoblastic leukemia; acute myeloid leukemia; chronic lymphocytic leukemia; chronic myeloid leukemia; I do not know; Other. | T0-T3 |
| 4a | In which year were you first diagnosed with the cancer for which you are receiving CAR T-cell therapy? | Year. | T0 |
| 5 | Have you ever previously been diagnosed with another cancer? | Yes; No; Free-text comment/specification of cancer. | T0 |
| 6 | What is your marital/relationship status? | Single; In a stable partnership/married; Divorced/partnership dissolved; Widowed. | T0-T3 |
| 7 | What is your highest educational qualification? | No school-leaving qualification; special school/primary or lower secondary school certificate; university of applied sciences entrance qualification; intermediate secondary school certificate; university entrance qualification; other educational qualification. | T0 |
| 8 | What is your employment status? | Student/pupil; employed; self-employed; homemaker; unemployed; retired/early retired; unable to work, percentage specified; other employment status. | T0-T3 |
| 9a | How many children do you have? | Number of children. | T0 |
| 9b | How old are your children? | Ages in years; free-text field. | T0 |

**B. Supportive care preferences and psychoeducation**

| **No.** | **English item wording** | **Response format / options** | **Timepoints** |
| --- | --- | --- | --- |
| 10 | Which of the following online-delivered support services would you use if they were available to you? | Multiple selection: individual psycho-oncological support; group psycho-oncological support; couple sessions; family sessions; participation in a support group for patients receiving CAR T-cell therapy; psychoeducation about symptoms that can occur in psychologically stressful situations and their treatment; social-law counseling; stress-reduction exercises, e.g., mindfulness-based stress reduction; presentation of assistive-device support and referral to individual counseling; information about financial support options; appointment for art therapy; appointment for music therapy; appointment for physiotherapy; instructions for physiotherapeutic exercises; nutrition tips; hygiene tips; assistive-device counseling; referral to a cancer counseling center; none; other. | T0-T3 |
| 11 | What amount of online-based psycho-oncological support would you prefer? | 1-5 sessions; 6-10 sessions; more than 10 sessions; no support; free-text comment/reason. | T0-T3 |
| 12 | Do you consider it useful to receive psychotherapeutic or psycho-oncological counseling and support after your inpatient treatment? | Yes; No; free-text comment/reason. | T0-T3 |
| 13 | Would long waiting times for psycho-oncological therapy appointments affect or burden you? | Yes; No; free-text comment/reason. | T0-T3 |
| 14 | Do you also consider it useful to offer psychotherapeutic or psycho-oncological counseling and support to your relatives or close caregivers? | Yes; No; free-text comment/reason. | T0-T3 |
| 15 | About which psychoeducational topics would you like to learn more? | Information about the cancer disease; information about cancer treatments; information about social support; information about emotional support and relief; information about stress management; information about problem-solving techniques; information about professional reorientation and development of new perspectives; other; free-text comment/reason. | T0-T3 |
| 16 | Which symptoms that may occur during CAR T-cell therapy do you currently fear most? | Neurological symptoms/neurotoxicity/confusional states; cytokine release syndrome/cytokine release/flu-like symptoms; allergic reactions; other. | T0-T3 |

**C. Knowledge, information preferences, and distress**

| **No.** | **English item wording** | **Response format / options** | **Timepoints** |
| --- | --- | --- | --- |
| 17a | Please circle the number from 0 to 10 that best describes how you rate your personal knowledge about your disease. | Numeric rating scale from 0 to 10. | T0-T3 |
| 17b | Please circle the number from 0 to 10 that best describes how you rate your personal knowledge about your therapy. | Numeric rating scale from 0 to 10. | T0-T3 |
| 18 | How would you like to receive new information about your disease or therapy? | Medical publications in specialist language; medical publications in plain language; explanatory videos; other; free-text comment/reason. | T0-T3 |
| 19 | Please circle the number from 0 to 10 that best describes how distressed you felt during the past 7 days. | Numeric rating scale from 0 to 10; 0 = not distressed at all; 10 = extremely distressed. | T0-T3 |

**D. Symptoms, coping, relational support, and digital intervention preferences**

| **No.** | **English item wording** | **Response format / options** | **Timepoints** |
| --- | --- | --- | --- |
| 20 | Are you currently experiencing increased difficulty relaxing? | Yes; No; free-text comment/reason. | T0-T3 |
| 21 | Would you participate in scientifically evaluated online interventions developed to help you relax, such as mindfulness-based stress reduction, progressive muscle relaxation, autogenic training, or guided imagery? | Yes; No; free-text comment/reason. | T0-T3 |
| 22 | Which stress- and symptom-reduction exercises would you like to participate in via an online tool if stress-related symptoms occurred? | Breathing exercises; guided imagery; progressive muscle relaxation; autogenic training; meditation; low-intensity yoga exercises; more demanding yoga exercises; music therapy; art therapy; other; free-text comment/reason. | T0-T3 |
| 23 | Are you currently experiencing increased problems falling asleep? | Yes; No; free-text comment/reason. | T0-T3 |
| 24 | Do you regularly take medication to help you fall asleep or sleeping tablets? | Yes; No; I do not know/medication reconciliation does not match; free-text comment/reason. | T0-T3 |
| 25 | Would you participate in scientifically evaluated online interventions developed to help you fall asleep? | Yes; No; free-text comment/reason. | T0-T3 |
| 26 | Are you currently experiencing increased anxiety? | Yes; No; free-text comment/reason. | T0-T3 |
| 27 | Would you participate in scientifically evaluated online interventions developed to make coping with anxiety easier? | Yes; No; free-text comment/reason. | T0-T3 |
| 28 | Do you have the opportunity to speak freely about your fears and worries with your partner or a close relative? | Yes; No; free-text comment/reason. | T0-T3 |
| 29 | Do you have the opportunity to speak freely about your illness and therapy with your partner or a close relative? | Yes; No; free-text comment/reason. | T0-T3 |
| 30 | Are you currently experiencing increased problems with low mood or depression? | Yes; No; free-text comment/reason. | T0-T3 |
| 31 | Would you participate in scientifically evaluated online interventions developed to help you cope with low mood or depression? | Yes; No; free-text comment/reason. | T0-T3 |
| 32 | Are you currently feeling increasingly left alone or lonely? | Yes; No; free-text comment/reason. | T0-T3 |
| 33 | Would you use online offers to reduce loneliness, such as referral to contact with other patients or support groups? | Yes; No; free-text comment/reason. | T0-T3 |
| 34 | Do you consider it useful to establish online contact with patients who have already completed CAR T-cell therapy? | Yes; No; free-text comment/reason. | T0-T3 |
| 35 | What further wishes do you have regarding possible online contact for information exchange, for example about symptoms and experiences with symptom-coping strategies, between patients receiving CAR T-cell therapy? | Open-ended free-text response. | T0-T3 |

**E. Psychiatric and psychosomatic history**

| **No.** | **English item wording** | **Response format / options** | **Timepoints** |
| --- | --- | --- | --- |
| 36 | Are you aware of any psychiatric diagnoses in yourself? | No; depression; psychosis; anxiety disorder; obsessive-compulsive disorder; bipolar disorder; alcohol misuse; trauma-related disorder; other. | T0-T3 |
| 37 | Have you ever been treated in a psychiatric or psychosomatic hospital? | Yes, inpatient; yes, outpatient; no; free-text comment/reason. | T0-T3 |
| 38a | Have you ever taken medication for a mental or psychosomatic illness? | Yes; No. | T0-T3 |
| 38b | For what purpose did you take the medication? | Not known; medication/purpose specified; question not answered. | T0-T3 |
| 38c | Which medication did you take? | Free-text medication name; question not answered. | T0-T3 |

**F. Understanding of the CAR T-cell treatment pathway**

| **No.** | **English item wording** | **Response format / options** | **Timepoints** |
| --- | --- | --- | --- |
| 39 | After the information session about CAR T-cell therapy, did you understand which steps now lie ahead of you? | Yes; No. | T0-T3 |
| 40 | Please describe which information from the information session you can still remember. | Open-ended free-text response. | T0-T3 |
| 41 | Please describe what you understand by the term leukapheresis. | Open-ended free-text response. | T0-T3 |
| 42 | Please describe what you understand by the term bridging therapy. | Open-ended free-text response. | T0-T3 |
| 43 | Please describe what you understand by the term conditioning chemotherapy. | Open-ended free-text response. | T0-T3 |

**G. Problem checklist during the past 7 days**

Question 44. Please indicate whether you worried about any of the following areas during the past 7 days or whether any of the following areas caused problems for you during the past 7 days. Please mark Yes or No for each area.

Response format for each area: Yes; No; no answer/not returned coded administratively where applicable.

| **Domain** | **Areas/items** |
| --- | --- |
| Practical problems | Housing; insurance; work; transportation; childcare. |
| Family problems | Relationship with partner; relationship with children. |
| Emotional problems | Worries; fears; sadness; depression; nervousness. |
| Spiritual/religious concerns | Concerns related to God; loss of faith. |
| Physical problems | Pain; nausea; exhaustion/fatigue; sleep; mobility; washing/dressing; physical appearance; breathing; mouth inflammation; eating/nutrition; digestive problems; constipation; diarrhea; changes in urination; fever; dry/itchy skin; dry/congested nose; tingling in hands/feet; feeling swollen/bloated; sexual problems. |
